# Sex-specific frailty and chronological age normative carotid artery intima-media thickness values using the Canadian longitudinal study of aging

**DOI:** 10.1177/17085381231157125

**Published:** 2023-02-14

**Authors:** Myles W O’Brien, Derek S Kimmerly, Olga Theou

**Affiliations:** 1 Division of Kinesiology, School of Health and Human Performance, 3688 Dalhousie University, Halifax, NS, Canada; 2 School of Physiotherapy (Faculty of Health) and Division of Geriatric Medicine (Faculty of Medicine), 3688 Dalhousie University, Halifax, NS, Canada; 3 Geriatric Medicine Research, 3688 Dalhousie University and Nova Scotia Health, Halifax, NS, Canada

**Keywords:** Arterial injury, frailty index, cardiovascular aging, Canadian Longitudinal Study of Aging

## Abstract

**Objectives:**

Carotid intima-media-thickness (cIMT) is predictive of future cardiovascular events, increases with chronological age, and greater in males. The accumulation of health deficits (or frailty) is a marker of biological age. However, normative cIMT values are lacking and would be an important comparative tool for healthcare providers and researchers. This study aimed to establish sex-specific normative cIMT values across chronological age and frailty levels (i.e. biological age).

**Methods:**

Frailty and right common cIMT data were extracted from the Canadian Longitudinal Study of Aging baseline comprehensive cohort of middle-aged and older adults (*n* = 10,209; 5000 females). cIMT was assessed via high-resolution ultrasound. Frailty was determined using a 52-item frailty index. Ordinary least squares and quantile regressions were conducted between age (years or frailty index) with cIMT (average or maximum), separately for males and females.

**Results:**

In both sexes, average and maximum cIMT increased with higher chronological age and frailty. Both cIMT metrics increased non-linearly (quadratic-cIMT term) with advancing age (β-coefficients for quadratic and linear terms: all, *p* < 0.001), except for the linear relationship between average and maximum cIMT with chronological age among males (*p* < 0.001). Sex-specific normative average and maximum cIMT values were established (1^st^–99^th^ percentiles, 5% increments), separately for chronological and biological ages.

**Conclusions:**

This is the largest sample of adults to establish normative cIMT outcomes that includes older adults. The chronological age and frailty-related normative cIMT outcomes will serve as a useful resource for healthcare professionals and researchers to establish “normal” age- and sex-specific cIMT values.

## Introduction

Cardiovascular diseases are among the leading causes of mortality worldwide.^1^ An increased thickening of arterial walls is characteristic of atherosclerosis and a major contributor toward the development of an adverse cardiovascular event.^2^ Thickness of common carotid artery intima-media layers (cIMT) may be non-invasively measured using high-resolution ultrasound,^3^ are a surrogate for arterial injury,^4^ and are proportionally associated with cardiovascular disease risk.^5^ Therefore, cIMT may have clinical utility.

It is well-established that cIMT increases with chronological age (i.e. age in years), at a rate of ∼0–40 μm per year.^6,7^ Sex differences in average cIMT have been documented, with values generally lower in middle-aged and older females versus males.^8^ Given the utility of average cIMT (i.e. typical outcome measure) and maximum cIMT (i.e. index of plaque development), understanding what a “normal” value is based on chronological age and sex has clinical importance. While average thickness is the more common cIMT outcome assessed, maximum cIMT is reliable, reproducible, and is postulated to provide a better index of atherosclerosis.^9^ As reviewed elsewhere,^10^ 22 cohort studies investigated percentiles of cIMT and observed that normative values may be region-specific, preventing the amalgamation of cohort studies. Importantly, most studies (*n* = 21/22) were based on groups of <7000 people, with most comprised of <4000 participants (16/22 studies),^10^ except for the Atherosclerosis Risk in Community study^11^ that presented average and maximum normative cIMT values in 13,610 (7463 females) middle-aged adults only. No study has been conducted to examine cIMT normative values in Canadian middle-aged or older adults. Including larger groups of participants (e.g. ∼10,000) could provide normative cIMT values that are more representative of the adult patient population.

While chronological age is the most used definition of aging, individuals of the same age may exhibit divergent biological states or “biological ages.” Frailty is multi-dimensional, provides a measure of vulnerability to health deficits,^12^ and has been promoted as a proxy for biological age.^13,14^ Frailty level (or biological age) may be more informative than chronological age and has been implemented as a routine measurement in some healthcare settings (e.g. geriatrics).^14^ In Canada, the prevalence of at least a mild level of frailty is 34% and 28% among middle-aged females and males, respectively.^15^ Frailty levels are typically higher in females versus males, despite lower frailty-related mortality in females.^16^ A multicenter clinical trial has demonstrated that a health deficit-based measure of frailty level is a key risk factor for cardiovascular morbidity and mortality.^17^ Across males and females, cIMT is positively associated with frailty level.^18^ Normative data for cIMT between males and females has been exclusively studied using chronological age,^10^ with no study investigating normative average or maximum cIMT values across percentiles of frailty (biological age).

The Canadian Longitudinal Study of Aging (CLSA) is a national random sample of Canadians aged 45–85 years that also includes a physical examination visit involving high-resolution ultrasound measured cIMT.^19^ The purpose of this study was to establish CLSA-based, sex-specific normative reference values for cIMT across chronological ages and frailty levels (proxy for biological age).

## Methods

The CLSA project has a comprehensive component consisting of questionnaires, a physical examination, and biological samples.^20,21^ Participants (chronological age: 45–85 years) are recruited across 7 provinces who live within a 50 km radius of one of the 11 data collection sites. The baseline data of participants who completed the physical examination (2012–2015) were used to answer the proposed research question (initially, *n* ∼ 30,000).

Participants who could not read and speak either French or English were excluded from the CLSA. As well, residents in the three territories, persons living on federal First Nations reserves, full-time members of the Canadian Armed Forces, persons with cognitive impairment at the time of recruitment and institutionalized individuals (e.g. living in long-term care facilities) were excluded. Descriptive characteristics are collected for the CLSA and are presented, including height, weight, body mass index, resting blood pressure, and resting heart rate.

Our protocol was approved by the CLSA Data and Sample Access Committee. As a matter of policy, the Nova Scotia Health Authority Research Ethics Board does not review research involving secondary analyses of datasets that contain deidentified individual-level data.

The frailty index implemented in the present analysis was based off of the deficit accumulation model and initially developed using the CLSA dataset,^15^ using standard procedures.^22^ The index was based on 52 items with each being coded as 0 (no deficit) or 1 (deficit). Interval or ordinal variables were coded as a proportion of complete deficit (e.g. self-rated health has 5 options: excellent = 0, very good = 0.25, good = 0.5, fair = 0.75, poor = 1). The frailty index was then calculated as the deficit score ÷ the number of deficits measured for each participant (e.g. 15/52 = 0.29), with a value closer to 1.00 indicating a higher degree of frailty.

Participant rested in the supine position for at least 5 min prior to measurements of the right common carotid artery with their head positioned at a 45° angle to the left. All images were obtained using a multi-frequency linear array probe attached to a high-resolution ultrasound system (Vivid i, General Electric Healthcare, Mississauga, ON, Canada) with concurrent electrocardiogram gating. The artery was imaged immediately proximal to the carotid bulb (external and internal carotid artery bifurcation). The application was pre-set for the 12 L probe, at a frequency of 11 MHz, depth of 4 cm, with a focal point positioned as close to the far wall of the carotid artery. Once an acceptable posterior cIMT image was observed (i.e. clear intima-media layers), a 3 cardiac cycle cineloop was recorded. Only clear intima-media layers were included in the region of interest for analysis, avoiding obvious plaques or areas of lower image quality. Average, maximum, and minimum cIMTs were measured. Average and maximum cIMTs have the most clinical relevance, given the predictive capabilities of average cIMT on cardiovascular disease risk^5^ and that the presence of atherosclerotic plaque results in higher maximum cIMT values. The right cIMT was utilized due to the greater number of assessments performed on the right versus left side, and coincides with recommendations that only a single observation is required due to the similarity between right and left cIMTs.^23^ For the present study, only participants with complete right side cIMT and frailty index data^15^ were included (Final: *n* = 10 209).

Although cIMT values were measured by different sonographers, training was provided, and a universal standard operating procedure was utilized for this nationwide study. As well, CLSA performed quality assurance in which 10 images were randomly selected each month, rated and further training was provided to individual staff if required. The standard operating procedures for all CLSA derived measurements are openly available (https://www.clsa-elcv.ca/researchers/physical-assessments). Only “good” quality images, and not images labeled as “re-analyzable” or “not usable” by the trained scanners were used. The specific process for labeling images is unclear, but concerns of “re-analyzable” images do not apply to this study as we omitted such files.

Participants’ characteristics were stratified by sex and are presented as means ± standard deviations. Descriptive characteristics were compared between males and females via independent samples *t*-tests.

Separate ordinary least squares regression analyses were conducted on the average and maximum cIMT variables. For each, the relationship of the cIMT variables with chronological age (predictor variable) or frailty index (measure of biological age; predictor variable) were determined separately for males and females. To explore whether the relationships were non-linear, alternative models were explored and included either: (1) linear and quadratic terms, or (2) linear, quadratic, and cubic terms. Except for chronological age in males, all other models were non-linear (i.e. significant quadratic predictor term). Sampling weights were applied to all regressions models to ensure national representations and to compensate for under-represented groups. We used the trimmed inflation weights, to report population-representative estimates following CLSA procedures.

Participants were grouped into 5-year intervals (45–49 years, 50–54, etc.) to determine normative values for chronological age. For biological age, participants were grouped into frailty levels of 0.05 intervals (e.g. 0.16–0.209, ≥0.21). Although, a level of 0.31 has been defined as moderate frailty level,^15^ there were too few participants above 0.26 or 0.31 to create its own category (see Table 1 for breakdown). Normative values were derived from quantile regressions of chronological age and frailty index (5^th^ percentile intervals from 5^th^ to 95^th^ percentiles, as well as 1^st^ and 99^th^ percentile) with average or maximum cIMT. Quantile regression models the relationship between predictor (independent) variables and specific percentiles (or quantiles) of the median for a target variable (i.e. cIMT).

**Table 1.** Participant characteristics, right common carotid intima-media thickness parameters, and frailty index.

Variable	Females (*n* = 5000)	Males (*n* = 5209)
***Participant characteristics***		
Age (years)	61.9 ± 10.2	62.3 ± 10.2
Age: 45–49 (*n*; %)	536 (10.7%)	548 (10.5%)
Age: 50–54 (*n*; %)	876 (17.5%)	916 (17.6%)
Age: 55–59 (*n*; %)	881 (17.6%)	793 (15.2%)
Age: 60–64 (*n*; %)	832 (16.6%)	921 (17.7%)
Age: 65–69 (*n*; %)	668 (13.4%)	702 (13.6%)
Age: 70–74 (*n*; %)	434 (8.7%)	487 (9.4%)
Age: 75–79 (*n*; %)	458 (9.2%)	524 (10.1%)
Age: 80–85 (*n*; %)	313 (6.3%)	316 (6.1%)
Height (meters)	1.62 ± 0.06	1.76 ± 0.07*
Body Mass (kilograms)	71 ± 15	86 ± 15*
Body mass index (kg/m^2^)	27.1 ± 5.5	27.7 ± 4.3*
Heart rate (beats/min)	72 ± 11	70 ± 12*
Systolic blood pressure (mmHg)	119 ± 17	122 ± 16*
Diastolic blood pressure (mmHg)	72 ± 9	76 ± 10*
***Right common carotid artery***		
Average cIMT (mm)	0.70 ± 0.14	0.73 ± 0.16*
Maximum cIMT (mm)	0.91 ± 0.20	0.95 ± 0.22*
Minimum cIMT (mm)	0.51 ± 0.14	0.54 ± 0.15*
***Frailty level***		
Frailty index (ratio)	0.089 ± 0.057	0.080 ± 0.054*
Level: 0.00–0.059 (*n*; %)	1831 (36.6%)	2224 (42.7%)
Level: 0.06–0.109 (*n*; %)	1641 (32.8%)	1681 (32.3%)
Level: 0.11–0.159 (*n*; %)	948 (19.0%)	853 (16.4%)
Level: 0.16–0.209 (*n*; %)	394 (7.9%)	306 (5.9%)
Level: ≥0.21 (*n*; %)	186 (3.7%)	145 (2.8%)

Data presented as means ± standard deviations or proportions (%). cIMT, carotid intima-media thickness. Frailty index was determined using a 52 variable based deficit accumulation model as the ratio between the number of deficits reported to the number of total variables. Results compared males and females via Independent Samples *t*-test. *p < 0.05 difference between males and females.

All statistics were completed in SPSS Version 28.0 (IBM, NY). Statistical significance was accepted as *p* < 0.05.

## Results

We analyzed a total of 5000 females and 5209 males with complete data. The number of male and female participants stratified by chronological age and frailty level are presented in Table 1. In general, males tended to be taller, heavier, have a higher blood pressure, but lower heart rate than females (all, *p* < 0.001). In the pooled sample, chronological age was not different between males and females (*p* = 0.10) On average, males had a thicker average, minimum, and maximum cIMT, but a lower frailty level than females (all, *p* < 0.001).

**Figure 1.**
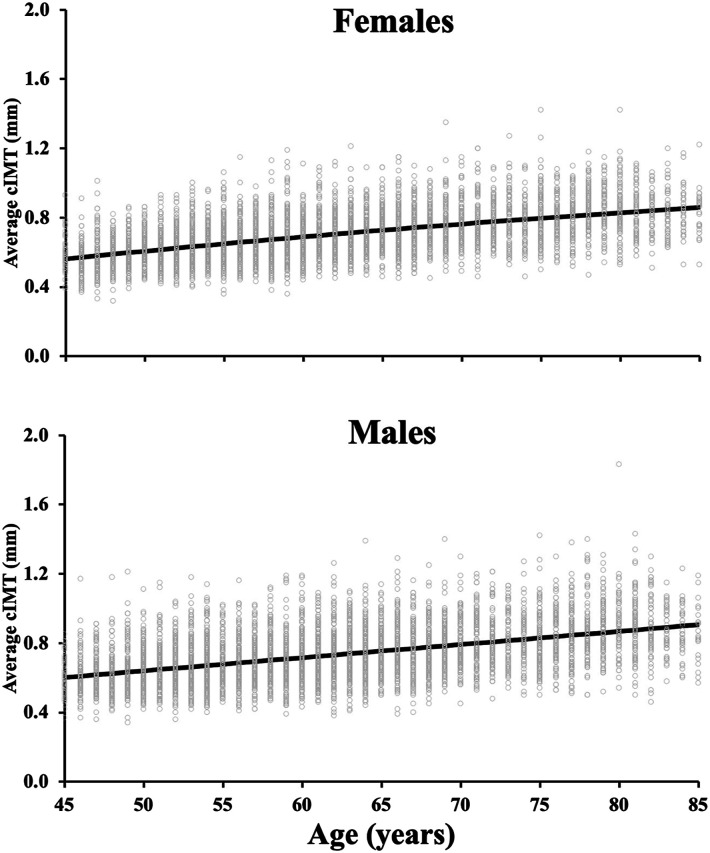
The relationship between chronological age and average right common carotid intima-media thickness (cIMT) among females (n = 5000; top panel) and males (n = 5209; bottom panel). The regression line is presented for both females (non-linear relationship) and males (linear relationship). The quadratic term for females is relatively small, (quadratic-β: −4.5 × 10−5^−5^), making the regression line appear linear in panel A, but it is non-linear. Individual participant values are presented. Ordinary least square regressions evaluated the relationship between cIMT and age for each sex.

In both sexes, older chronological age was associated with a larger average cIMT (Figure 1, Tables 2 and 3) and maximum cIMT (Supplemental Tables 1–2). Among females, the relationship between chronological age and average cIMT was curvilinear (adjusted *R*^2^ = 0.30, linear-β: 0.013 [95% CI: 0.009–0.017], quadratic-β: −4.5 × 10^−5^ [−7.6 × 10^−5^, −1.4 × 10^−5^], both, *p* < 0.001). In females, this curvilinear relationship remained for maximum cIMT (adjusted *R*^2^ = 0.21, *p* < 0.001). However, this relationship was linear for average cIMT (adjusted *R*^2^ = 0.21) and maximum cIMT (adjusted *R*^2^ = 0.15) among males (all, *p* < 0.005; Figure 1). The cIMT reference values from the 1^st^ to 99^th^ percentiles (5% intervals) are presented for average cIMT (Tables 2 and 3) and maximum cIMT (Supplemental Tables 1–2), separately for males and females.

**Table 2.** Normative average carotid intima-media thickness values based on chronological age for middle-aged and older males.

Percentile	Males age group (years)							
	45–49	50–54	55–59	60–64	65–69	70–74	75–79	80–85
**1**	0.40	0.44	0.44	0.46	0.50	0.53	0.52	0.53
**5**	0.47	0.48	0.50	0.52	0.56	0.59	0.61	0.61
**10**	0.49	0.51	0.54	0.55	0.59	0.64	0.65	0.68
**15**	0.51	0.54	0.56	0.58	0.62	0.66	0.68	0.71
**20**	0.52	0.56	0.58	0.60	0.64	0.68	0.71	0.74
**25**	0.54	0.57	0.60	0.62	0.66	0.70	0.74	0.78
**30**	0.55	0.58	0.61	0.64	0.68	0.73	0.76	0.80
**35**	0.57	0.60	0.62	0.66	0.70	0.74	0.78	0.82
**40**	0.58	0.62	0.64	0.68	0.71	0.75	0.80	0.84
**45**	0.59	0.63	0.66	0.70	0.73	0.77	0.82	0.85
**50**	0.60	0.65	0.68	0.72	0.75	0.80	0.84	0.87
**55**	0.62	0.66	0.69	0.73	0.78	0.81	0.86	0.89
**60**	0.63	0.68	0.71	0.75	0.80	0.84	0.88	0.91
**65**	0.65	0.70	0.72	0.77	0.82	0.86	0.89	0.94
**70**	0.67	0.72	0.75	0.79	0.84	0.90	0.92	0.96
**75**	0.69	0.74	0.77	0.82	0.86	0.92	0.94	0.99
**80**	0.71	0.77	0.80	0.85	0.88	0.94	0.97	1.02
**85**	0.75	0.80	0.83	0.88	0.91	0.97	1.00	1.05
**90**	0.79	0.84	0.87	0.93	0.96	1.01	1.05	1.09
**95**	0.85	0.92	0.94	0.99	1.01	1.08	1.13	1.16
**99**	0.97	1.04	1.07	1.12	1.15	1.20	1.30	1.30

Carotid intima-media thickness collected in the right common carotid artery (posterior edge) using Brightness-mode ultrasound presented in millimeters.

**Table 3.** Normative average carotid intima-media thickness values based on chronological age for middle-aged and older females.

Percentile	Females age group (years)							
	45–49	50–54	55–59	60–64	65–69	70–74	75–79	80–85
**1**	0.38	0.43	0.44	0.48	0.48	0.52	0.54	0.53
**5**	0.43	0.48	0.49	0.52	0.56	0.59	0.59	0.63
**10**	0.47	0.51	0.52	0.56	0.59	0.62	0.64	0.67
**15**	0.49	0.53	0.54	0.58	0.62	0.65	0.68	0.69
**20**	0.50	0.54	0.56	0.60	0.64	0.67	0.70	0.71
**25**	0.52	0.56	0.58	0.62	0.65	0.69	0.73	0.74
**30**	0.53	0.57	0.60	0.64	0.67	0.71	0.74	0.76
**35**	0.54	0.58	0.61	0.65	0.69	0.72	0.76	0.78
**40**	0.55	0.59	0.62	0.66	0.70	0.73	0.78	0.79
**45**	0.56	0.60	0.64	0.68	0.73	0.75	0.80	0.82
**50**	0.57	0.62	0.65	0.70	0.74	0.77	0.81	0.83
**55**	0.58	0.63	0.66	0.71	0.76	0.79	0.82	0.85
**60**	0.59	0.64	0.68	0.73	0.77	0.81	0.84	0.86
**65**	0.61	0.66	0.69	0.74	0.79	0.83	0.86	0.87
**70**	0.62	0.67	0.71	0.76	0.80	0.85	0.88	0.90
**75**	0.64	0.69	0.73	0.78	0.82	0.87	0.91	0.92
**80**	0.66	0.70	0.76	0.80	0.84	0.90	0.94	0.94
**85**	0.69	0.73	0.78	0.82	0.87	0.93	0.96	0.98
**90**	0.72	0.76	0.83	0.85	0.90	0.95	1.00	1.02
**95**	0.77	0.81	0.88	0.90	0.96	1.03	1.04	1.07
**99**	0.86	0.91	1.02	1.04	1.09	1.12	1.13	1.18

Carotid intima-media thickness collected in the right common carotid artery (posterior edge) using Brightness-mode ultrasound presented in millimeters.

**Figure 2.**
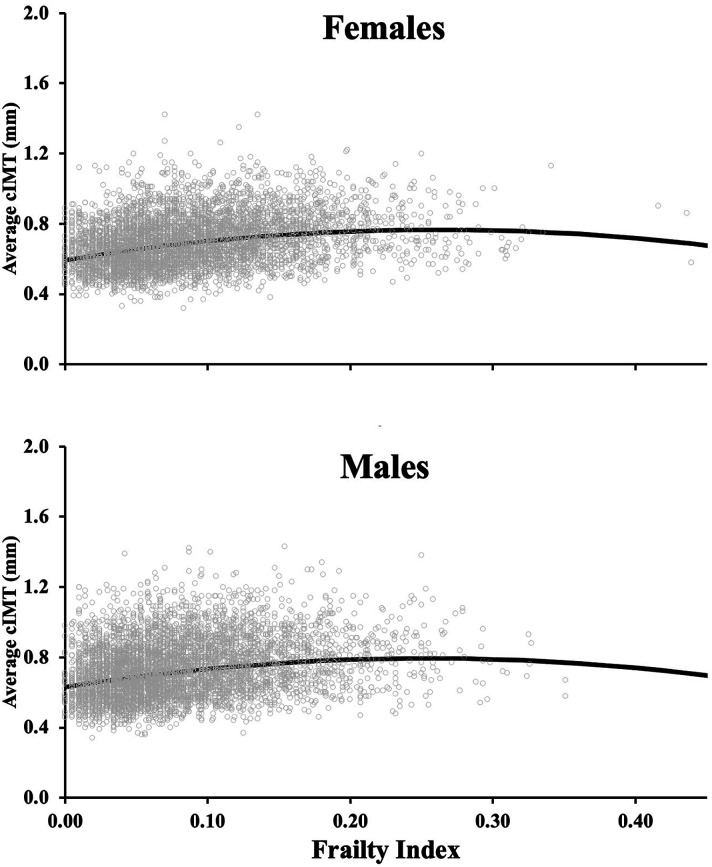
The relationship between frailty level (marker of biological age) and average right common carotid intima-media thickness (cIMT) among females (n = 5000; top panel) and males (n = 5209; bottom panel). The regression line is presented for both females (non-linear relationship) and males (non-linear relationship). Individual participant values are presented. Frailty index was determined using a 52 variable based deficit accumulation model as the ratio between the number of deficits reported to the number of total variables. Ordinary least square regressions evaluated the relationship between cIMT and frailty level for each sex.

Unlike chronological age, which demonstrated a more gradual positive relationship between age and cIMT (Figure 1; albeit quadratic for females), the slope for the relationship between frailty level (proxy of biological age) with cIMT was more curvilinear (Figure 2; quadratic β-values: −2.5 vs. −4.5 × 10^−5^). Both males and females demonstrated a non-linear relationship between frailty and either average or maximum cIMT (β-coefficients for quadratic and linear terms: all, *p* < 0.001). For females, the relationship average cIMT-frailty: adjusted *R*^2^ = 0.10, linear-β: 1.3 [95% CI: 1.1–1.5], quadratic-β: −2.5 [−3.3, −1.8], all *p* < 0.001). For males, the relationship average cIMT-frailty: adjusted *R*^2^ = 0.06, linear-β: 1.3 [95% CI: 1.0–1.5], quadratic-β: −2.5 [−3.6, −1.4], all *p* < 0.001). The curvilinear relationship for both sexes rapidly increased between frailty levels of 0–∼0.21 and then leveled off or slightly decreased beyond this frailty level (Figure 2). For frailty, reference values from the 1^st^–99^th^ percentiles (5% intervals) are presented for average cIMT (Tables 4 and 5) and maximum cIMT (Supplemental Tables 3-4), separately for males and females.

**Table 4.** Normative average carotid intima-media thickness values based on a frailty index for middle-aged and older males.

	Male frailty index (ratio)				
Percentile	0.00–0.059	0.06–0.109	0.11–0.159	0.16–0.209	≥0.21
**1**	0.43	0.46	0.48	0.50	0.49
**5**	0.49	0.52	0.54	0.56	0.56
**10**	0.52	0.56	0.58	0.60	0.60
**15**	0.54	0.58	0.61	0.63	0.63
**20**	0.56	0.61	0.64	0.65	0.68
**25**	0.58	0.63	0.66	0.69	0.70
**30**	0.60	0.65	0.68	0.71	0.71
**35**	0.62	0.67	0.70	0.72	0.75
**40**	0.64	0.69	0.73	0.76	0.77
**45**	0.66	0.71	0.75	0.78	0.80
**50**	0.67	0.72	0.77	0.80	0.81
**55**	0.69	0.75	0.79	0.82	0.83
**60**	0.71	0.77	0.82	0.84	0.85
**65**	0.73	0.80	0.84	0.86	0.88
**70**	0.76	0.82	0.87	0.88	0.89
**75**	0.78	0.85	0.90	0.91	0.92
**80**	0.81	0.88	0.93	0.95	0.94
**85**	0.84	0.92	0.96	0.98	0.96
**90**	0.89	0.96	1.00	1.03	1.05
**95**	0.96	1.03	1.07	1.07	1.11
**99**	1.12	1.18	1.20	1.29	1.38

Carotid intima-media thickness collected in the right common carotid artery (posterior edge) using Brightness-mode ultrasound presented in millimeters. Frailty index was determined using a 52 variable based deficit accumulation model as the ratio between the number of deficits reported to the number of total variables. Frailty index is an index of biological age.

**Table 5.** Normative average carotid intima-media thickness values based on a frailty index for middle-aged and older females.

	Female frailty index (ratio)				
Percentile	0.00–0.059	0.06–0.109	0.11–0.159	0.16–0.209	≥0.21
**1**	0.43	0.43	0.46	0.48	0.49
**5**	0.48	0.50	0.53	0.55	0.57
**10**	0.50	0.54	0.57	0.58	0.61
**15**	0.53	0.56	0.59	0.61	0.63
**20**	0.55	0.58	0.62	0.64	0.65
**25**	0.57	0.60	0.64	0.67	0.66
**30**	0.58	0.62	0.66	0.70	0.68
**35**	0.59	0.64	0.68	0.72	0.70
**40**	0.61	0.66	0.70	0.74	0.71
**45**	0.62	0.67	0.72	0.76	0.73
**50**	0.64	0.69	0.74	0.78	0.76
**55**	0.65	0.70	0.76	0.79	0.77
**60**	0.67	0.73	0.77	0.81	0.80
**65**	0.69	0.75	0.79	0.82	0.83
**70**	0.70	0.77	0.81	0.85	0.85
**75**	0.73	0.79	0.84	0.87	0.86
**80**	0.75	0.81	0.86	0.90	0.91
**85**	0.78	0.85	0.89	0.93	0.95
**90**	0.82	0.88	0.93	0.97	0.99
**95**	0.87	0.94	0.99	1.04	1.04
**99**	1.02	1.09	1.10	1.15	1.15

Carotid intima-media thickness collected in the right common carotid artery (posterior edge) using Brightness-mode ultrasound presented in millimeters. Frailty index was determined using a 52 variable based deficit accumulation model as the ratio between the number of deficits reported to the number of total variables. Frailty index is an index of biological age.

## Discussion

Using a large sample of middle-aged and older Canadian adults, this study established sex-specific normative values for average and maximum right common cIMT across chronological and biological (via frailty) definitions of aging. We document the non-linear associations between cIMT with frailty and age among females, and positive linear age-cIMT relationship in males. These outcomes may be a useful reference for healthcare providers and researchers to compare their patients cIMT against a reference to determine carotid artery health.

A thicker cIMT is indicative of arterial injury^4^ and generally worse cardiovascular health.^24^ It should be acknowledged that the clinical utility of cIMT measurements has been debated^7^ and may provide less cardiovascular risk insight than a coronary artery calcium scores.^25^ However, a recent high level meta-analysis of 119 clinical randomized controlled trials demonstrated a proportional relationship between the magnitude of cIMT and cardiovascular disease risk.^5^ Accordingly, healthcare providers and researchers may benefit from measuring patients’ cIMT as part of a comprehensive cardiovascular health assessment. Specifically, knowing whether patients’ cIMT is abnormally high relative to their sex-specific chronological or biological age could be useful for characterizing their health risk and prompting interventional strategies that to put them on a healthier aging trajectory. While normative values have been established in a variety of populations outside of Canada,^6,26^ they have been primarily in smaller samples of adults and have produced heterogeneous results.^10^ While the Atherosclerosis Risk in Community study^11^ also included >10,000 participants, their sample was comprised of middle-aged adults only. Certainly, older adults (>65 years of age) exhibit larger cIMT and have worse cardiovascular health than middle-aged adults.^27^ This study adds to the current literature by including the largest sample of participants that includes older adults. Therefore, we were able to establish normative values stratified by sex across a wide age range of Canadian middle-aged and older adults. Importantly, we included both average cIMT (i.e. typical outcome measure^5^) and maximum cIMT (i.e. index of plaque development^9^). While average thickness is the more common cIMT outcome presented, maximum cIMT is postulated to provide a better index of atherosclerosis.^9^ The reference values calculated herein may serve as an important resource for healthcare providers and researchers to determine whether their patients exhibit normal cIMT values as a function of their age. As reviewed elsewhere, cohorts generally observe maximal cIMT values that range from 0.72 to 1.22 mm, with most ∼0.95 mm for adults 60–70 years.^10^ These values are generally consistent with the maximum cIMTs in the present study (50^th^ percentile: ∼0.88–0.96 mm for 60–70 years). This reference may help identify whether patients exhibit an abnormally large cIMT as a function of their age and sex, and therefore indicates poor arterial health that may warrant medical tests/procedures or prompt lifestyle interventions.

As two individuals of the same age may present very differently in terms of health status, it is argued that biological age may be more informative for health than chronological age.^14^ Frailty level describes the vulnerability to health deficits^12^ and may reflect biological age. Accordingly, the measurement of frailty level is becoming more common within healthcare settings.^28^ While frailty level increases with chronological age,^15^ the relationship with cIMT tends to differ between chronological age and frailty (see Figure 1 versus Figure 2). Within the milder frailty levels (0.00–0.21), there was a steeper relationship between frailty level and thicker cIMT, which leveled off at higher levels of frailty ∼0.21 (moderately frail) for both sexes (see Figure 2). This is possibly due to our frailty index being a general indicator of vulnerability to adverse health outcomes,^29^ and unfavorable health deficits other than cardiovascular function (e.g. mental health, vision, etc.) are impacting these more frail individuals. Accordingly, the normative values for cIMT may be less useful for people living with high degrees of frailty. However, such people may have numerous other health concerns that may surpass the need to establish their normative cIMT value. Alternatively, it could be due to having relatively few individuals with frailty levels >0.26 (*n* = 80 total), making the establishment of normative values for such high frailty levels, challenging (Table 1). Nevertheless, individuals with frailty levels <0.21 comprise most of the population (∼96% of the sample; see Table 1) and the frailty normative values established herein useful in clinical settings that routinely measure frailty level. The inclusion of a frailty index as a proxy of biological age in our study may promote the future development of normative values as a function of physiological age in addition to chronological age among other areas of study (e.g. cognition, other vascular measures, etc.) to understand normative outcomes beyond simply years of age.

This study is strengthened by its large representative sample of middle-aged and older males and females. Accordingly, this is the largest study to establish age- and sex-specific normative values for cIMT. The variability in measurements within operators or between assessment sites (11 across Canada) was not determined but all sites adopted a strict standard operating procedure. The CLSA excludes adults older than 85 years at entry into the study, persons with cognitive impairments, and those living in long-term care facilities. These persons may exhibit worse cardiovascular health and/or higher levels of frailty level (e.g. >0.31), and therefore the results of this study should not be extrapolated to these populations.

## Conclusion

A higher chronological age and higher frailty levels are associated with a larger average and maximum cIMT among males and females. This is consistently non-linear for females, but age-cIMT is linear for males. This large CLSA dataset may serve as a reference for Canadian healthcare providers and researchers to compare their patients’ cIMT to the population as a function of chronological and/or biological age.

## Supplemental Material

Supplemental Material - Sex-specific frailty and chronological age normative carotid artery intima-media thickness values using the Canadian longitudinal study of aging Supplemental Material for Sex-specific frailty and chronological age normative carotid artery intima-media thickness values using the Canadian longitudinal study of aging by Myles W O’Brien, Derek S Kimmerly,and Olga Theou in Vascular

Supplemental Material - Sex-specific frailty and chronological age normative carotid artery intima-media thickness values using the Canadian longitudinal study of aging Supplemental Material for Sex-specific frailty and chronological age normative carotid artery intima-media thickness values using the Canadian longitudinal study of aging by Myles W O’Brien, Derek S Kimmerly,and Olga Theou in Vascular

## References

[1] Barquera S Pedroza-Tobías A Medina C , et al. Global overview of the epidemiology of atherosclerotic cardiovascular disease. Arch Med Res 2015; 46: 328–338. 26135634 10.1016/j.arcmed.2015.06.006

[2] Anand SS Yusuf S Vuksan V , et al. Differences in risk factors, atherosclerosis, and cardiovascular disease between ethnic groups in Canada: the study of health assessment and risk in ethnic groups (SHARE). Lancet 2000; 356: 279–284. 11071182 10.1016/s0140-6736(00)02502-2

[3] Gaarder M Seierstad T . Measurements of carotid intima media thickness in non-invasive high-frequency ultrasound images: the effect of dynamic range setting. Cardiovasc Ultrasound 2015; 13: 5. 25628215 10.1186/1476-7120-13-5 PMC4328984

[4] Raggi P Stein JH . Carotid intima-media thickness should not be referred to as subclinical atherosclerosis: a recommended update to the editorial policy at atherosclerosis. Atherosclerosis 2020; 312: 119–120. 32994032 10.1016/j.atherosclerosis.2020.09.015

[5] Willeit P Tschiderer L Allara E , et al. Carotid intima-media thickness progression as surrogate marker for cardiovascular risk: meta-Analysis of 119 clinical trials involving 100 667 patients. Circulation 2020; 142: 621–642. 32546049 10.1161/CIRCULATIONAHA.120.046361 PMC7115957

[6] Pastorius CA Medina-Lezama J Corrales-Medina F , et al. Normative values and correlates of carotid artery intima-media thickness and carotid atherosclerosis in andean-hispanics: the prevencion study. Atherosclerosis 2010; 211: 499–505. 20510418 10.1016/j.atherosclerosis.2010.04.009 PMC2928715

[7] Lorenz MW Polak JF Kavousi M , et al. Carotid intima-media thickness progression to predict cardiovascular events in the general population (the PROG-IMT collaborative project): a meta-analysis of individual participant data. Lancet 2012; 379: 2053–2062. 22541275 10.1016/S0140-6736(12)60441-3 PMC3918517

[8] Łoboz-Rudnicka M Bociąga Z Jaroch J , et al. Impact of cardiovascular risk factors on carotid intima-media thickness: sex differences. Clin Interv Aging 2016; 11: 721–731. 27307718 10.2147/CIA.S103521 PMC4887056

[9] Bots ML Evans GW Riley WA , et al. Carotid intima-media thickness measurements in intervention studies: design options, progression rates, and sample size considerations: a point of view. Stroke 2003; 34: 2985–2994. 14615619 10.1161/01.STR.0000102044.27905.B5

[10] Liao X Norata GD Polak JF , et al. Normative values for carotid intima media thickness and its progression: are they transferrable outside of their cohort of origin? Eur J Prev Cardiol 2016; 23: 1165–1173. 26746227 10.1177/2047487315625543

[11] Nambi V Chambless L Folsom AR , et al. Carotid intima-media thickness and presence or absence of plaque improves prediction of coronary heart disease risk: the ARIC (atherosclerosis risk in communities) study. J Am Coll Cardiol 2010; 55: 1600–1607. 20378078 10.1016/j.jacc.2009.11.075 PMC2862308

[12] Mitnitski AB Mogilner AJ Rockwood K . Accumulation of deficits as a proxy measure of aging. Sci.World J 2001; 1: 323–336. 10.1100/tsw.2001.58 PMC6084020 12806071

[13] Li X Ploner A Wang Y , et al. Longitudinal trajectories, correlations and mortality associations of nine biological ages across 20-years follow-up. Elife 2020; 9: e51507. Epub ahead of print 11 February 2020. DOI: 10.7554/eLife.51507 32041686 PMC7012595

[14] Diebel LWM Rockwood K . Determination of biological age: Geriatric assessment vs biological biomarkers. Curr Oncol Rep 2021; 23: 104. 34269912 10.1007/s11912-021-01097-9 PMC8284182

[15] Pérez-Zepeda MU Godin J Armstrong JJ , et al. Frailty among middle-aged and older Canadians: population norms for the frailty index using the Canadian longitudinal study on aging. Age Ageing 2021; 50: 447–456. 32805022 10.1093/ageing/afaa144

[16] Gordon EH Peel NM Samanta M , et al. Sex differences in frailty: a systematic review and meta-analysis. Exp Gerontol 2017; 89: 30–40. 28043934 10.1016/j.exger.2016.12.021

[17] Farooqi MAM Gerstein H Yusuf S , et al. Accumulation of deficits as a key risk factor for cardiovascular morbidity and mortality: a pooled analysis of 154 000 individuals. J Am Heart Assoc 2020; 9: e014686. Epub ahead of print 4 February 2020. DOI: 10.1161/JAHA.119.014686 31986990 PMC7033862

[18] O’Brien MW Kimmerly DS Theou O . Impact of age and sex on the relationship between carotid intima-media thickness and frailty level in the Canadian longitudinal study of aging. J Cardiol. In Press 2023. 10.1016/j.jjcc.2023.01.004 36682711

[19] Kirkland SA Griffith LE Menec V , et al. Mining a unique Canadian resource: the Canadian longitudinal study on aging. Can J Aging 2015; 34: 366–377. 26300192 10.1017/S071498081500029X

[20] Raina PS Wolfson C Kirkland SA , et al. The Canadian longitudinal study on aging (CLSA). Can J Aging/La Rev Can du Vieil 2009; 28: 221–229. 10.1017/S0714980809990055 19860977

[21] Raina P Wolfson C Kirkland S , et al. Cohort profile: the Canadian longitudinal study on aging (CLSA). Int J Epidemiol 2019; 48: 1752–1753. 31633757 10.1093/ije/dyz173 PMC6929533

[22] Searle SD Mitnitski A Gahbauer EA , et al. A standard procedure for creating a frailty index. BMC Geriatr 2008; 8: 24. 18826625 10.1186/1471-2318-8-24 PMC2573877

[23] Loizou CP Nicolaides A Kyriacou E , et al. A comparison of ultrasound intima-media thickness measurements of the left and right common carotid artery. IEEE J Transl Eng Health Med 2015; 3: 1–10. 10.1109/JTEHM.2015.2450735 PMC4848048 27170894

[24] Simon A Megnien J-L Chironi G . The value of carotid intima-media thickness for predicting cardiovascular risk. Arterioscler Thromb Vasc Biol 2010; 30: 182–185. 19948842 10.1161/ATVBAHA.109.196980

[25] Mach F Baigent C Catapano AL , et al. 2019 ESC/EAS guidelines for the management of dyslipidaemias: lipid modification to reduce cardiovascular risk. Atherosclerosis 2019; 290: 140–205. 31591002 10.1016/j.atherosclerosis.2019.08.014

[26] Ciccone MM Balbarini A Teresa Porcelli M , et al. Carotid artery intima-media thickness: normal and percentile values in the Italian population (camp study). Eur J Cardiovasc Prev Rehabil 2011; 18: 650–655. 21450588 10.1177/1741826711398841

[27] van den Munckhof ICL Jones H Hopman MTE , et al. Relation between age and carotid artery intima-medial thickness: a systematic review. Clin Cardiol 2018; 41: 698–704. 29752816 10.1002/clc.22934 PMC6489962

[28] Theou O Squires E Mallery K , et al. What do we know about frailty in the acute care setting? a scoping review. BMC Geriatr 2018; 18: 139. 29898673 10.1186/s12877-018-0823-2 PMC6000922

[29] Cesari M Gambassi G van Kan GA , et al. The frailty phenotype and the frailty index: different instruments for different purposes. Age Ageing 2014; 43: 10–12. 24132852 10.1093/ageing/aft160

