# Supplementary figures and images for: Sex-specific frailty and chronological age normative carotid artery intima-media thickness values using the Canadian longitudinal study of aging
Source: Vascular. 2023 Feb 14;32(3):579–88. doi: 10.1177/17085381231157125 (PMC11129517; doi:10.1177/17085381231157125)

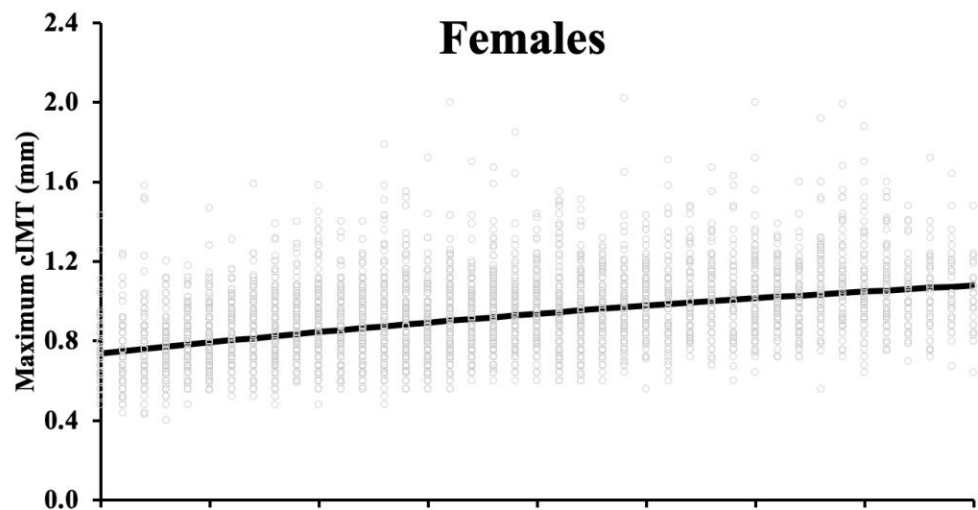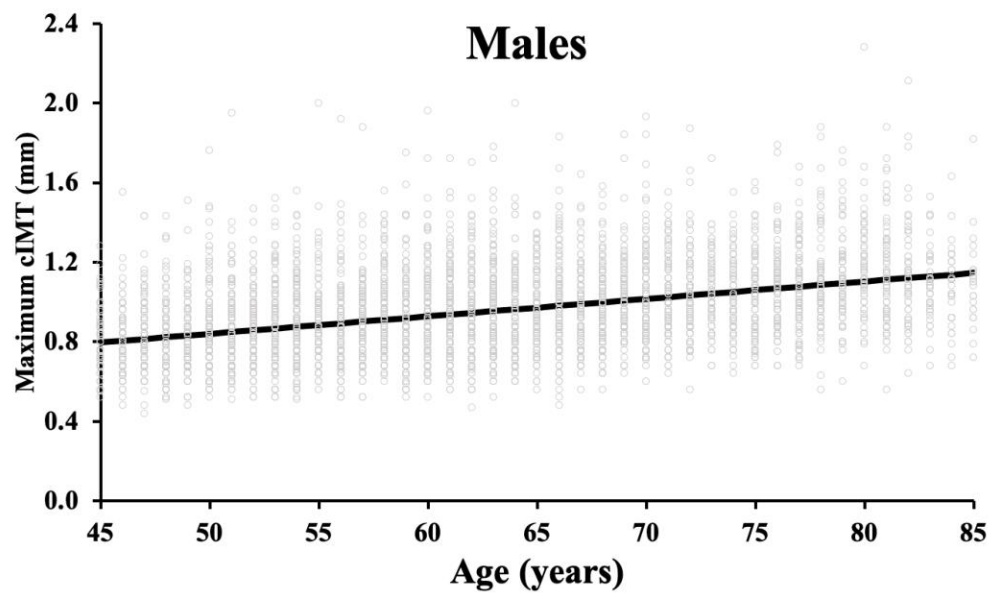

Supplement: Supplemental Material - Sex-specific frailty and chronological age normative carotid artery intima-media thickness values using the Canadian longitudinal study of aging [file sj-pdf-1-vas-10.1177_17085381231157125.pdf]

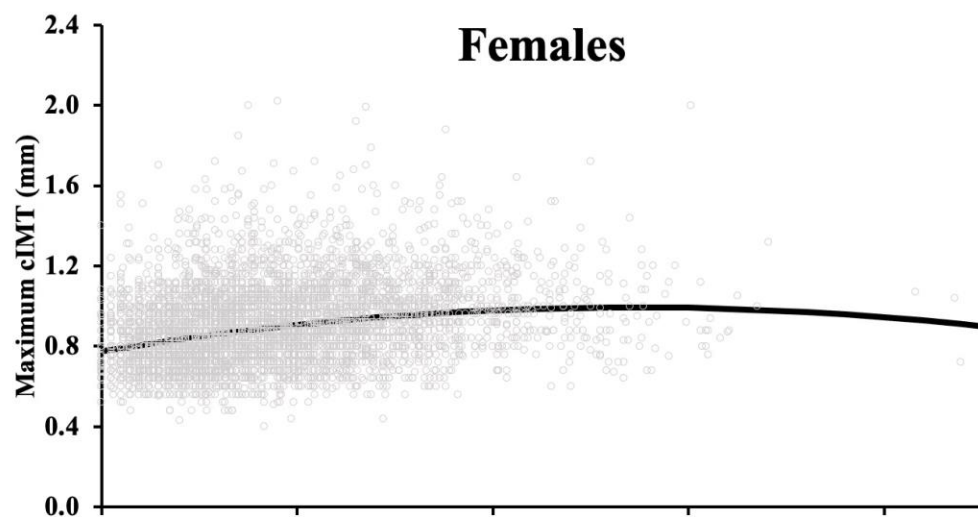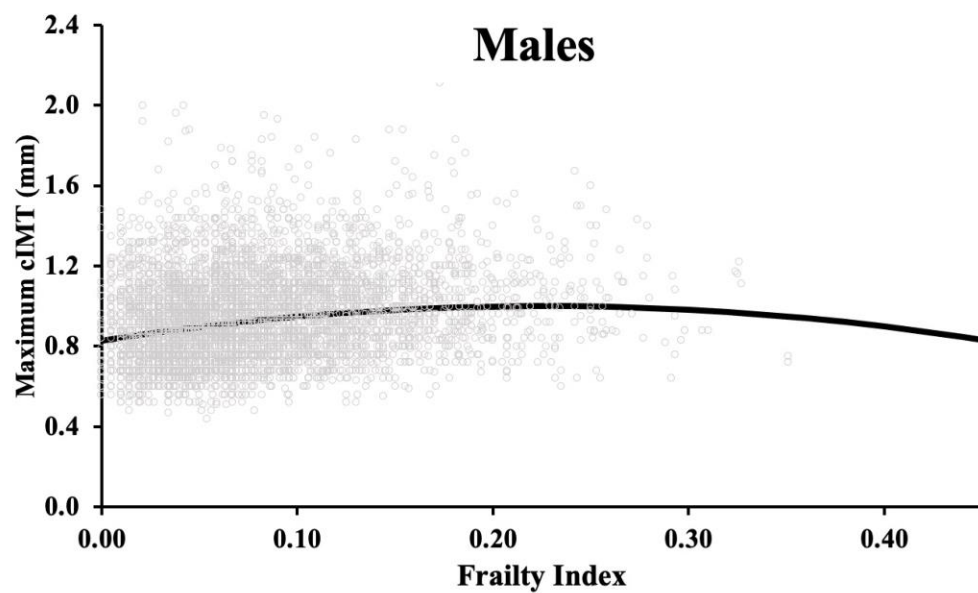

Supplement: Supplemental Material - Sex-specific frailty and chronological age normative carotid artery intima-media thickness values using the Canadian longitudinal study of aging [file sj-pdf-2-vas-10.1177_17085381231157125.pdf]
